# Dimethyl 2,5-bis­(5-hexyl­thio­phen-2-yl)benzene-1,4-dioate

**DOI:** 10.1107/S1600536811011718

**Published:** 2011-04-07

**Authors:** Cheng-Li Song, Ke Liu, Ai-Jiang Zhang, Zhu-Guo Xu, Hao-Li Zhang

**Affiliations:** a State Key Laboratory of Applied Organic Chemistry (SKLAOC), College of Chemistry and Chemical Engineering, Lanzhou University, Lanzhou, 730000, People’s Republic of China

## Abstract

In the title compound, C_30_H_38_O_4_S_2_, the centroid of the benzene ring lies on a center of inversion. The thio­phene ring is aligned at 49.8 (1)° with respect to the benzene ring. The alkyl chain adopts an extended zigzag conformation.

## Related literature

The title compound and its derivatives are used in the preparation of organic semiconductors. For applications of these materials, see: Tian *et al.* (2010 ▶); Zhang *et al.* (2010 ▶). For the synthesis of related compounds, see: Fraind & Tovar (2010 ▶); Gurthrie & Tovar (2008 ▶), Hotta (2001 ▶); Kang *et al.* (1997 ▶); Lois *et al.* (2007 ▶); Shao & Zhao (2009 ▶); Zhao *et al.* (2007 ▶). 
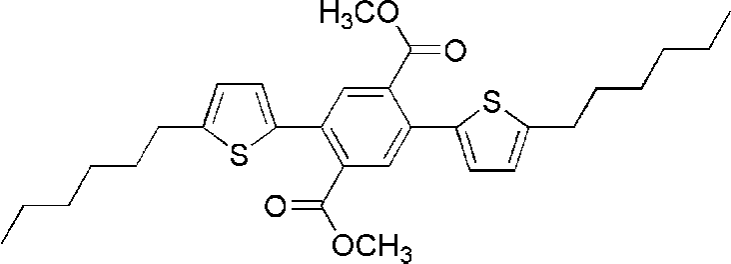


## Experimental

### 

#### Crystal data

C_30_H_38_O_4_S_2_
*M* *_r_* = 526.72
Monoclinic, 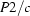

*a* = 15.617 (6) Å
*b* = 8.083 (3) Å
*c* = 11.585 (4) Å
β = 104.470 (4)°
*V* = 1416.0 (9) Å^3^
*Z* = 2
Mo *K*α radiation
μ = 0.22 mm^−1^
*T* = 293 K
0.35 × 0.32 × 0.19 mm

#### Data collection

Bruker APEXII CCD diffractometer
Absorption correction: multi-scan (*SADABS*; Bruker, 2005 ▶) *T*_min_ = 0.927, *T*_max_ = 0.959
6083 measured reflections
2490 independent reflections
1865 reflections with *I* > 2σ(*I*)
*R*_int_ = 0.031

#### Refinement

*R*[*F* ^2^ > 2σ(*F* ^2^)] = 0.041
*wR*(*F* ^2^) = 0.104
*S* = 1.04
2490 reflections
165 parameters
H-atom parameters constrained
Δρ_max_ = 0.16 e Å^−3^
Δρ_min_ = −0.23 e Å^−3^

### 

Data collection: *APEX2* (Bruker, 2005 ▶); cell refinement: *SAINT* (Bruker, 2005 ▶); data reduction: *SAINT*; program(s) used to solve structure: *SHELXS97* (Sheldrick, 2008 ▶); program(s) used to refine structure: *SHELXL97* (Sheldrick, 2008 ▶); molecular graphics: *SHELXTL* (Sheldrick, 2008 ▶); software used to prepare material for publication: *SHELXTL*.

## Supplementary Material

Crystal structure: contains datablocks I, global. DOI: 10.1107/S1600536811011718/ng5141sup1.cif

Structure factors: contains datablocks I. DOI: 10.1107/S1600536811011718/ng5141Isup2.hkl

Additional supplementary materials:  crystallographic information; 3D view; checkCIF report

## Figures and Tables

**Fig. 1.**
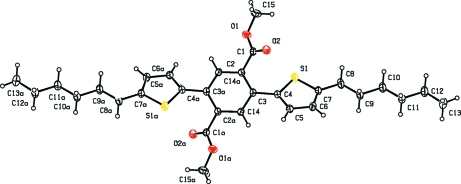
The molecular structure of the title compound. Displacement ellipsoids are drawn at the 30% probability level. Symmetry operations: a = -x, 1 - y, -z.

**Fig. 2.**
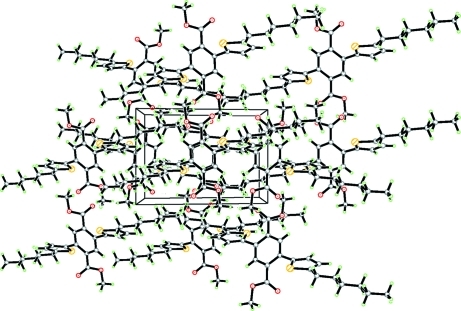
Molecular packing of the title compound (I).

## supplementary crystallographic information

### Comment

The title compound and its derivatives are important materials for the preparation of various organic semiconductors, which could find applications in the fields of organic light-emitting diodes (OLEDs), organic field-effect transistors (OFETs) or solar cells (Tian *et al.*, 2010; Zhang *et al.*, 2010). The device performance of these organic semiconductors are strongly dependent on the molecular packing in their crystals. This led us to pay attention to the synthesis and crystal structure of the compound. Herein, we report the synthesis and structure of the title compound, namely dimethyl 2,5-bis(5-hexylthiophen-2-yl)benzene-1,4-dioate.

The molecular structure of the title compound is shown in Fig.1. Bond lengths and angles in the molecule are within normal ranges. The bond length of C—H in the thiophene and the benzene rings is 0.93 Å and the angle formed by C—S—C in the thiophene is 92.79°. In this structure, the two thiophene rings and the benzene ring are not in the same plane, which give a dihedral angle of 49.84°.

### Experimental

The title compound was synthesized by Suzuki cross-coupling reaction. Briefly, thiophene-2-boronic acid (2.52 g, 10.00 mmol), dimethyl 2,5-dibromobenzene-1,4-dioate (880 mg, 2.50 mmol), Pd(PPh_3_)_4_ (115 mg, 0.10 mmol) and NaHCO_3_ (840 mg, 10.00 mmol) were mixed in dry THF (10 ml) under argon. The mixture was stirred and refluxed for 24 hrs (monitored by TLC) to give the title compound. The product was purified by column chromatography. Colorless crystals were grown via evaporation from n-hexane and dichloromethane (10:1, *v*:*v*) mixture solvents at room temperature for X-ray diffraction.

### Refinement

All H atoms were placed in geometrically idealized positions, with C—H = 0.93 Å, and constrained to ride on their respective parent atoms, with *U*_iso_(H) = 1.2Ueq(C).

### Crystal data

**Table:**

C_30_H_38_O_4_S_2_	*F*(000) = 564
*M**_r_* = 526.72	*D*_x_ = 1.235 Mg m^−^^3^
Monoclinic, *P*2/*c*	Melting point: 362 K
Hall symbol: -P 2yc	Mo *K*α radiation, λ = 0.71073 Å
*a* = 15.617 (6) Å	Cell parameters from 1616 reflections
*b* = 8.083 (3) Å	θ = 2.5–25.8°
*c* = 11.585 (4) Å	µ = 0.22 mm^−^^1^
β = 104.470 (4)°	*T* = 293 K
*V* = 1416.0 (9) Å^3^	Block, colorless
*Z* = 2	0.35 × 0.32 × 0.19 mm

### Data collection

**Table:**

Bruker APEXII CCD diffractometer	2490 independent reflections
Radiation source: fine-focus sealed tube	1865 reflections with *I* > 2σ(*I*)
graphite	*R*_int_ = 0.031
φ and ω scans	θ_max_ = 25.1°, θ_min_ = 2.5°
Absorption correction: multi-scan (*SADABS*; Bruker, 2005)	*h* = −16→18
*T*_min_ = 0.927, *T*_max_ = 0.959	*k* = −8→9
6083 measured reflections	*l* = −13→13

### Refinement

**Table:**

Refinement on *F*^2^	Primary atom site location: structure-invariant direct methods
Least-squares matrix: full	Secondary atom site location: difference Fourier map
*R*[*F*^2^ > 2σ(*F*^2^)] = 0.041	Hydrogen site location: inferred from neighbouring sites
*wR*(*F*^2^) = 0.104	H-atom parameters constrained
*S* = 1.04	*w* = 1/[σ^2^(*F*_o_^2^) + (0.0496*P*)^2^ + 0.1877*P*] where *P* = (*F*_o_^2^ + 2*F*_c_^2^)/3
2490 reflections	(Δ/σ)_max_ < 0.001
165 parameters	Δρ_max_ = 0.16 e Å^−^^3^
0 restraints	Δρ_min_ = −0.23 e Å^−^^3^

### Special details

**Table:**

Geometry. All e.s.d.'s (except the e.s.d. in the dihedral angle between two l.s. planes) are estimated using the full covariance matrix. The cell e.s.d.'s are taken into account individually in the estimation of e.s.d.'s in distances, angles and torsion angles; correlations between e.s.d.'s in cell parameters are only used when they are defined by crystal symmetry. An approximate (isotropic) treatment of cell e.s.d.'s is used for estimating e.s.d.'s involving l.s. planes.
Refinement. Refinement of *F*^2^ against ALL reflections. The weighted *R*-factor *wR* and goodness of fit *S* are based on *F*^2^, conventional *R*-factors *R* are based on *F*, with *F* set to zero for negative *F*^2^. The threshold expression of *F*^2^ > σ(*F*^2^) is used only for calculating *R*-factors(gt) *etc*. and is not relevant to the choice of reflections for refinement. *R*-factors based on *F*^2^ are statistically about twice as large as those based on *F*, and *R*- factors based on ALL data will be even larger.

### Fractional atomic coordinates and isotropic or equivalent isotropic displacement parameters (Å^2^)

**Table:**

	*x*	*y*	*z*	*U*_iso_*/*U*_eq_	
C1	0.07441 (12)	0.8250 (2)	−0.01209 (17)	0.0320 (5)	
C2	0.03976 (12)	0.6534 (2)	−0.00661 (17)	0.0301 (4)	
C3	0.07574 (12)	0.5472 (2)	0.08892 (16)	0.0309 (5)	
C4	0.15632 (12)	0.5867 (2)	0.18357 (17)	0.0329 (5)	
C5	0.16918 (13)	0.5716 (3)	0.30285 (18)	0.0411 (5)	
H5	0.1254	0.5364	0.3388	0.049*	
C6	0.25579 (14)	0.6143 (3)	0.36780 (19)	0.0457 (6)	
H6	0.2739	0.6121	0.4506	0.055*	
C7	0.30953 (12)	0.6585 (2)	0.29820 (18)	0.0364 (5)	
C8	0.40559 (13)	0.7071 (3)	0.3327 (2)	0.0480 (6)	
H8A	0.4122	0.8115	0.2946	0.058*	
H8B	0.4393	0.6246	0.3020	0.058*	
C9	0.44453 (13)	0.7245 (3)	0.4653 (2)	0.0472 (6)	
H9A	0.4376	0.6205	0.5037	0.057*	
H9B	0.4115	0.8080	0.4960	0.057*	
C10	0.54203 (14)	0.7720 (3)	0.4984 (2)	0.0504 (6)	
H10A	0.5750	0.6890	0.4672	0.060*	
H10B	0.5489	0.8764	0.4604	0.060*	
C11	0.58113 (14)	0.7884 (3)	0.6310 (2)	0.0539 (6)	
H11A	0.5466	0.8688	0.6621	0.065*	
H11B	0.5752	0.6830	0.6682	0.065*	
C12	0.67691 (15)	0.8399 (3)	0.6673 (2)	0.0581 (7)	
H12A	0.7115	0.7618	0.6342	0.070*	
H12B	0.6827	0.9475	0.6330	0.070*	
C13	0.71464 (18)	0.8491 (3)	0.7999 (2)	0.0792 (9)	
H13A	0.6816	0.9277	0.8334	0.119*	
H13B	0.7754	0.8833	0.8164	0.119*	
H13C	0.7111	0.7422	0.8345	0.119*	
C14	0.03448 (12)	0.3957 (2)	0.09348 (17)	0.0327 (5)	
H14	0.0574	0.3248	0.1569	0.039*	
C15	0.11166 (16)	1.0295 (3)	−0.1356 (2)	0.0549 (6)	
H15A	0.0682	1.1060	−0.1218	0.082*	
H15B	0.1181	1.0434	−0.2153	0.082*	
H15C	0.1673	1.0503	−0.0797	0.082*	
O1	0.08339 (9)	0.86154 (16)	−0.12051 (12)	0.0441 (4)	
O2	0.09033 (9)	0.91985 (17)	0.07033 (12)	0.0444 (4)	
S1	0.25288 (3)	0.65154 (7)	0.15037 (5)	0.0447 (2)	

### Atomic displacement parameters (Å^2^)

**Table:**

	*U*^11^	*U*^22^	*U*^33^	*U*^12^	*U*^13^	*U*^23^
C1	0.0258 (10)	0.0378 (11)	0.0301 (12)	−0.0019 (8)	0.0026 (9)	−0.0002 (9)
C2	0.0278 (10)	0.0345 (10)	0.0280 (11)	−0.0041 (8)	0.0071 (8)	−0.0029 (9)
C3	0.0264 (10)	0.0364 (11)	0.0283 (12)	−0.0025 (8)	0.0039 (8)	−0.0035 (9)
C4	0.0285 (10)	0.0340 (10)	0.0337 (12)	−0.0047 (8)	0.0031 (9)	−0.0004 (9)
C5	0.0347 (12)	0.0546 (13)	0.0321 (13)	−0.0116 (10)	0.0048 (9)	0.0031 (10)
C6	0.0422 (13)	0.0577 (14)	0.0311 (13)	−0.0109 (10)	−0.0025 (10)	0.0009 (10)
C7	0.0282 (10)	0.0383 (11)	0.0379 (13)	−0.0035 (9)	−0.0010 (9)	−0.0023 (9)
C8	0.0298 (11)	0.0531 (13)	0.0562 (16)	−0.0086 (10)	0.0017 (11)	−0.0064 (11)
C9	0.0319 (12)	0.0486 (13)	0.0535 (15)	−0.0053 (10)	−0.0032 (11)	−0.0030 (11)
C10	0.0336 (12)	0.0491 (13)	0.0603 (17)	−0.0045 (10)	−0.0037 (11)	−0.0043 (12)
C11	0.0404 (13)	0.0528 (14)	0.0580 (17)	−0.0087 (11)	−0.0072 (12)	0.0015 (12)
C12	0.0387 (13)	0.0582 (15)	0.0659 (18)	−0.0052 (11)	−0.0086 (12)	−0.0039 (13)
C13	0.0651 (18)	0.085 (2)	0.066 (2)	−0.0177 (15)	−0.0225 (15)	0.0089 (16)
C14	0.0298 (10)	0.0379 (11)	0.0279 (11)	−0.0003 (8)	0.0023 (9)	0.0011 (9)
C15	0.0672 (16)	0.0483 (14)	0.0546 (16)	−0.0134 (12)	0.0257 (13)	0.0083 (11)
O1	0.0577 (10)	0.0423 (9)	0.0349 (9)	−0.0128 (7)	0.0162 (7)	−0.0015 (6)
O2	0.0542 (9)	0.0409 (8)	0.0345 (9)	−0.0097 (7)	0.0039 (7)	−0.0071 (7)
S1	0.0315 (3)	0.0660 (4)	0.0355 (4)	−0.0110 (3)	0.0062 (2)	−0.0034 (3)

### Geometric parameters (Å, °)

**Table:**

C1—O2	1.201 (2)	C9—H9B	0.9700
C1—O1	1.331 (2)	C10—C11	1.510 (3)
C1—C2	1.496 (3)	C10—H10A	0.9700
C2—C14^i^	1.390 (3)	C10—H10B	0.9700
C2—C3	1.403 (3)	C11—C12	1.508 (3)
C3—C14	1.391 (3)	C11—H11A	0.9700
C3—C4	1.483 (2)	C11—H11B	0.9700
C4—C5	1.351 (3)	C12—C13	1.503 (3)
C4—S1	1.728 (2)	C12—H12A	0.9700
C5—C6	1.416 (3)	C12—H12B	0.9700
C5—H5	0.9300	C13—H13A	0.9600
C6—C7	1.349 (3)	C13—H13B	0.9600
C6—H6	0.9300	C13—H13C	0.9600
C7—C8	1.505 (3)	C14—C2^i^	1.390 (3)
C7—S1	1.721 (2)	C14—H14	0.9300
C8—C9	1.511 (3)	C15—O1	1.452 (2)
C8—H8A	0.9700	C15—H15A	0.9600
C8—H8B	0.9700	C15—H15B	0.9600
C9—C10	1.523 (3)	C15—H15C	0.9600
C9—H9A	0.9700		
			
O2—C1—O1	123.96 (18)	C11—C10—H10A	108.8
O2—C1—C2	124.28 (18)	C9—C10—H10A	108.8
O1—C1—C2	111.74 (16)	C11—C10—H10B	108.8
C14^i^—C2—C3	119.57 (16)	C9—C10—H10B	108.8
C14^i^—C2—C1	118.65 (16)	H10A—C10—H10B	107.7
C3—C2—C1	121.56 (16)	C12—C11—C10	115.4 (2)
C14—C3—C2	118.10 (17)	C12—C11—H11A	108.4
C14—C3—C4	118.50 (17)	C10—C11—H11A	108.4
C2—C3—C4	123.39 (17)	C12—C11—H11B	108.4
C5—C4—C3	128.22 (18)	C10—C11—H11B	108.4
C5—C4—S1	109.88 (14)	H11A—C11—H11B	107.5
C3—C4—S1	121.82 (14)	C13—C12—C11	114.0 (2)
C4—C5—C6	113.54 (19)	C13—C12—H12A	108.7
C4—C5—H5	123.2	C11—C12—H12A	108.7
C6—C5—H5	123.2	C13—C12—H12B	108.7
C7—C6—C5	113.65 (19)	C11—C12—H12B	108.7
C7—C6—H6	123.2	H12A—C12—H12B	107.6
C5—C6—H6	123.2	C12—C13—H13A	109.5
C6—C7—C8	129.7 (2)	C12—C13—H13B	109.5
C6—C7—S1	110.14 (15)	H13A—C13—H13B	109.5
C8—C7—S1	120.20 (16)	C12—C13—H13C	109.5
C7—C8—C9	114.55 (19)	H13A—C13—H13C	109.5
C7—C8—H8A	108.6	H13B—C13—H13C	109.5
C9—C8—H8A	108.6	C2^i^—C14—C3	122.33 (18)
C7—C8—H8B	108.6	C2^i^—C14—H14	118.8
C9—C8—H8B	108.6	C3—C14—H14	118.8
H8A—C8—H8B	107.6	O1—C15—H15A	109.5
C8—C9—C10	113.74 (19)	O1—C15—H15B	109.5
C8—C9—H9A	108.8	H15A—C15—H15B	109.5
C10—C9—H9A	108.8	O1—C15—H15C	109.5
C8—C9—H9B	108.8	H15A—C15—H15C	109.5
C10—C9—H9B	108.8	H15B—C15—H15C	109.5
H9A—C9—H9B	107.7	C1—O1—C15	115.35 (16)
C11—C10—C9	113.8 (2)	C7—S1—C4	92.78 (10)
			
O2—C1—C2—C14^i^	129.1 (2)	C5—C6—C7—S1	1.1 (2)
O1—C1—C2—C14^i^	−49.3 (2)	C6—C7—C8—C9	−6.6 (3)
O2—C1—C2—C3	−45.5 (3)	S1—C7—C8—C9	174.20 (15)
O1—C1—C2—C3	136.11 (18)	C7—C8—C9—C10	179.42 (19)
C14^i^—C2—C3—C14	−0.7 (3)	C8—C9—C10—C11	−179.62 (19)
C1—C2—C3—C14	173.85 (17)	C9—C10—C11—C12	−178.4 (2)
C14^i^—C2—C3—C4	178.01 (17)	C10—C11—C12—C13	−177.9 (2)
C1—C2—C3—C4	−7.4 (3)	C2—C3—C14—C2^i^	0.7 (3)
C14—C3—C4—C5	−48.0 (3)	C4—C3—C14—C2^i^	−178.05 (17)
C2—C3—C4—C5	133.3 (2)	O2—C1—O1—C15	−2.0 (3)
C14—C3—C4—S1	128.25 (17)	C2—C1—O1—C15	176.42 (16)
C2—C3—C4—S1	−50.5 (2)	C6—C7—S1—C4	−0.48 (17)
C3—C4—C5—C6	177.57 (18)	C8—C7—S1—C4	178.84 (17)
S1—C4—C5—C6	0.9 (2)	C5—C4—S1—C7	−0.28 (16)
C4—C5—C6—C7	−1.4 (3)	C3—C4—S1—C7	−177.16 (16)
C5—C6—C7—C8	−178.1 (2)		

Symmetry codes: (i) −*x*, −*y*+1, −*z*.
